# Concordance Between Electronic Health Record Data and Medicare Part D Claims Data for Oral Anticancer Drug Use

**DOI:** 10.1001/jamanetworkopen.2020.3821

**Published:** 2020-04-30

**Authors:** Manvi Sharma, Michael L. Johnson, Hui Zhao, Sharon H. Giordano, Holly M. Holmes

**Affiliations:** 1 Department of Pharmacy Administration, School of Pharmacy, The University of Mississippi, Oxford; 2 Department of Pharmaceutical Health Outcomes and Policy, College of Pharmacy, University of Houston, Houston, Texas; 3 Department of Health Services Research, The University of Texas MD Anderson Cancer Center, Houston; 4 Division of Geriatric and Palliative Medicine, McGovern Medical School, The University of Texas Health Science Center, Houston

## Abstract

This cross-sectional study determines the concordance between electronic health record and Medicare Part D claims data for the receipt of oral anticancer agents.

## Introduction

Real-world evidence from electronic health records (EHRs) and claims data are being evaluated for use in regulatory decision-making.^1,2^ The objective of our study was to determine the concordance between EHR and Medicare Part D (MPD) claims data for the receipt of oral anticancer agents, a rapidly growing treatment option for cancer.

## Methods

In this cross-sectional study, MPD claims were linked with EHRs for patients treated at The University of Texas MD Anderson Cancer Center (MDACC) via the Texas Cancer registry. The institutional review boards of The University of Texas MDACC, the Texas Cancer Registry, and the Centers for Medicare & Medicaid Services approved this study. Informed consent was waived because this study described all data in aggregate form, without the identification of any individual participants, and involved no more than minimal risk to participants. Furthermore, the waiver would not adversely affect the rights and welfare of the participants, and the research could not be carried out without the waiver. This study followed the Strengthening the Reporting of Observational Studies in Epidemiology (STROBE) reporting guideline for cross-sectional studies.

Patients were aged 65 years and older; had breast, prostate, kidney, or colon cancer or chronic myeloid leukemia; and were enrolled in MPD during treatment at MDACC between January 2007 and December 2012. Use of any oral anticancer agent was extracted from the MDACC EHR through retrospective medical record review of reconciled medication lists, pharmacy records, and clinic notes (eFigure in the Supplement). The EHR did not have computerized order entry, and thus, the data reflect medication reconciliation, indicating potential use of a medication. Event files in MPD claims data were searched to identify claims for any oral anticancer drug for each patient and to reflect prescriptions that were actually dispensed to patients.

Data analysis was conducted from November 2017 to April 2019, in SAS Enterprise Guide 6.1 (SAS Institute). We ascertained EHR and MPD concordance rates with κ statistics after matching drug name and requiring overlapping treatment dates. No prespecified level of significance was set.

## Results

The study sample consisted of 208 EHR medication records and 250 MPD claims for 170 patients. Patients had a median (interquartile range) age of 69 (65-73) years, and 106 (62.4%) were men. There were 22 different oral anticancer drugs evaluated (Box). Bicalutamide (74 records [29.6%] in MPD, 54 records [25.9%] in EHR), anastrozole (36 [14.4%] in MPD, 34 [16.3%] in EHR), and pazopanib (25 [10.0%] in MPD, 18 [8.6%] in EHR) were the most frequently used drugs. The overall percentage agreement between the 2 data sets was 73.8%, in which 176 events were yes in both data sets and 123 events were no in both data sets. The percentage disagreement was 26.2%, in which 74 MPD claims (18.3%) were not found in the EHR, and 32 EHR drugs (7.9%) were not found in the MPD (κ = 0.47; 95% CI, 0.39-0.56) (Table).

Box. **List of Oral Anticancer Drugs Assessed for Use**

1. Abiraterone
2. Anastrozole
3. Axitinib
4. Bicalutamide
5. Dasatinib
6. Diethyl Stilbestrol
7. Enzalutamide
8. Erlotinib
9. Estramustine
10. Everolimus
11. Exemestane
12. Flutamide
13. Hydroxyurea
14. Imatinib
15. Letrozole
16. Nilotinib
17. Nilutamide
18. Pazopanib
19. Sorafenib
20. Sunitinib
21. Tamoxifen
22. Thalidomide

**Table.** Comparison of Oral Anticancer Drug Claims Between Electronic Health Records and Medicare Part D

Oral anticancer drugs in electronic health records	Oral anticancer drugs in Medicare Part D claims		
	Yes	No	Total
Yes	176	32	208
No	74	123	197
Total	250	155	405

## Discussion

The moderate rate of agreement (ie, 73.8%) found in this study may be because of various reasons. The EHR may be missing oral anticancer use for patients receiving only a consultation or because of incomplete medication reconciliation or medical record information. Claims in MPD may be missing because patients could obtain medications by other means that do not result in an MPD claim, including discount or other assistance programs. Du et al^3^ reported a 96% rate of agreement with a κ of 0.72 (95% CI, 0.64-0.79) between a tumor registry and medical records for tamoxifen or aromatase-inhibitors for breast cancer. Lund et al^4^ reported 55% sensitivity and 47% specificity of Medicare claims to identify the use of capecitabine when compared with data from the National Cancer Institute Patterns of Care study. However, to our knowledge, our study was the first to compare MPD claims with EHR data for use of oral anticancer agents. A potential limitation of this study is that it was a single site study, thus the results may not be generalizable to all MPD claims.

This study provides an estimate of the potential information difference that may be present when EHR or claims data alone are used, with important implications for studies of oral anticancer drug use patterns, drug spending, outcomes, and quality measures. For the drugs that are in the EHR but are missing from MPD (8%), there is concern regarding how to fully evaluate the use of costly anticancer drugs using MPD data.^2,5^ For the drugs that are in the MPD but are missing in EHR (18%), there is concern regarding the completeness of utilization patterns that are generated based on EHR.^6^

A data linkage between EHR and administrative claims would potentially improve the capture of exposures and outcomes and thus produce better quality data that could be used in regulatory decision-making.

## Notes

Supplement. **eFigure.** Detailed Methods for the Study and the Process of Manual Chart Review
