# Supplementary material for: Concordance Between Electronic Health Record Data and Medicare Part D Claims Data for Oral Anticancer Drug Use
Source: JAMA Netw Open. 2020 Apr 30;3(4):e203821. doi: 10.1001/jamanetworkopen.2020.3821 (PMC7193329; doi:10.1001/jamanetworkopen.2020.3821)
Supplement: Supplement. — eFigure. Detailed Methods for the Study and the Process of Manual Chart Review [file jamanetwopen-3-e203821-s001.pdf]

Supplementary Online Content

Sharma M, Johnson ML, Zhao H, Giordano SH, Holmes HM. Concordance between electronic health record data and Medicare Part D claims data for oral anticancer drug use. *JAMA Netw Open*. 2020;3(4):e203821. doi:10.1001/jamanetworkopen.2020.3821

eFigure. Detailed Methods for the Study and the Process of Manual Chart Review

This supplementary material has been provided by the authors to give readers additional information about their work.

© 2020 Sharma M et al. *JAMA Network Open.*

eFigure. Detailed Methods for the Study and the Process of Manual Chart Review

Patient selection: 1230 patients were identified in the MDACC system that met the initial inclusion criteria of age≥65, diagnosis of breast, prostate, kidney, colon cancer or Chronic Myeloid Leukemia, treatment at MDACC during the study period of January 2007 to December 2012 and having Medicare benefits. 623 patients were included in screening based on *a priori* sample size calculations. Medicare Part D enrollment dates for patients were identified. 170 patients with overlapping Medicare Part D enrollment and MDACC medication use dates constituted the study sample.

Chart abstraction form: The data collection form was designed and tested on 10 patients by two independent data abstractors. After a few minor modifications, the abstract form was finalized. The complete data for 170 patients was extracted by one reviewer. The reviewer was not blinded to the study objectives. Complete EHR data extraction was done before linking the records with the Part D claims to maintain consistency of data extraction.

Chart abstraction process: Medication use was determined in the EHR in three places:

Reconciled Medication List. Any record of an oral anti-cancer drug in the medication list was noted in the data abstraction sheet. The dates from this reconciled medication list also served as medication use dates for the patient. The medication use dates were used for matching with Medicare Part D enrollment dates for patients. If there were no current medications listed and clinic notes confirmed that the patient received treatment outside MDACC, then the indicated patient was considered not treated at MDACC and was excluded from the study.

Pharmacy Records. The “pharmacy records” were the records for the internal hospital pharmacy, which included inpatient and outpatient medications dispensed to patients. Any record of oral anti-cancer drug in this section was noted in the data abstraction sheet.

Clinic Notes. The “clinic notes” were the transcribed clinic notes that were reviewed for confirmation of the oral anti-cancer drug use or lack of use. Any record of oral anti-cancer drug was noted in the data abstraction sheet.

Medicare Part D events: A crosswalk for the Medicare patient IDs was obtained from Texas Cancer Registry and was used to link the Medicare Part D claims data for patients treated at MDACC that were included in the EHR data abstraction. Any record of oral anti-cancer drug from Part D event files for the included patients was identified. Records from Part D files indicated that a prescription was dispensed to a patient.

Statistical Analysis: The baseline characteristics for the patients were measured. The concordance rates were ascertained after matching the drug names for each patient. Kappa statistic was calculated to assess the concordance. All the analysis was carried out using SAS Enterprise Guide 6.1 (SAS Institute, Cary, NC).

The data collection and analysis were conducted from November 2017 through April 2019. The institutional review boards of The University of Texas MD Anderson Cancer Center, Texas Cancer Registry and Centers for Medicare and Medicaid Services approved this study. Informed consent was waived because this research study

© 2020 Sharma M et al. *JAMA Network Open.*

described all data in aggregate form only without the identification of any individual participants and involved no more than minimal risk to subjects and the waiver would not adversely affect the rights and welfare of the subjects and the research could not practicable carried out without the waiver. This study followed the STROBE reporting guideline for cross-sectional studies.

© 2020 Sharma M et al. *JAMA Network Open.*
